# Supplementary material for: The Construction and Evaluation of a Multi-Task Convolutional Neural Network for a Cone-Beam Computed-Tomography-Based Assessment of Implant Stability
Source: Diagnostics (Basel). 2022 Nov 3;12(11):2673. doi: 10.3390/diagnostics12112673 (PMC9689694; doi:10.3390/diagnostics12112673)
Supplement: Supplementary file 1 [file diagnostics-12-02673-s001.zip › diagnostics-1986949-supplementary.pdf]

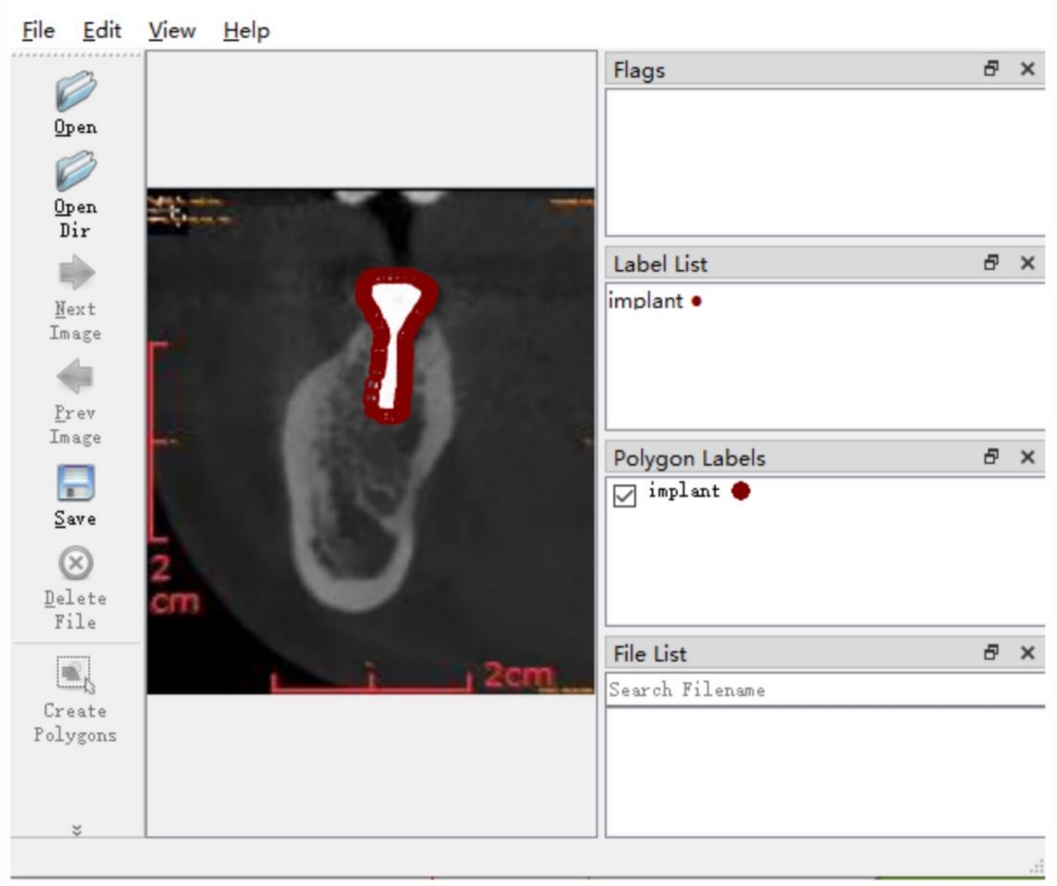

Figure S1. The image labeling process using the data annotation software LabelMe.

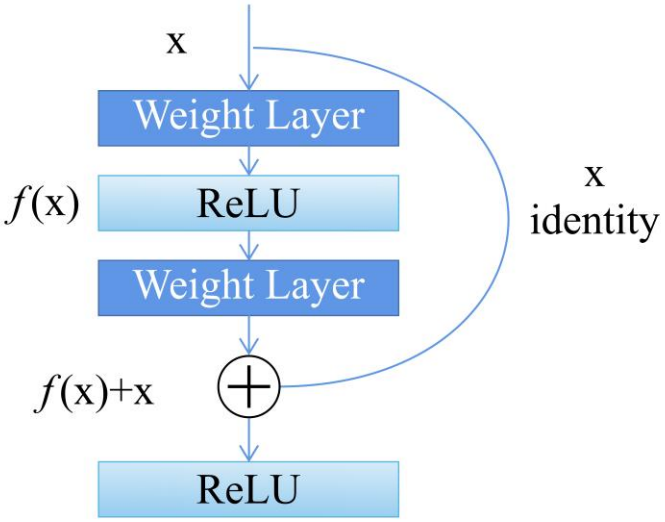

Figure S2. Schematic diagram of residual structure.

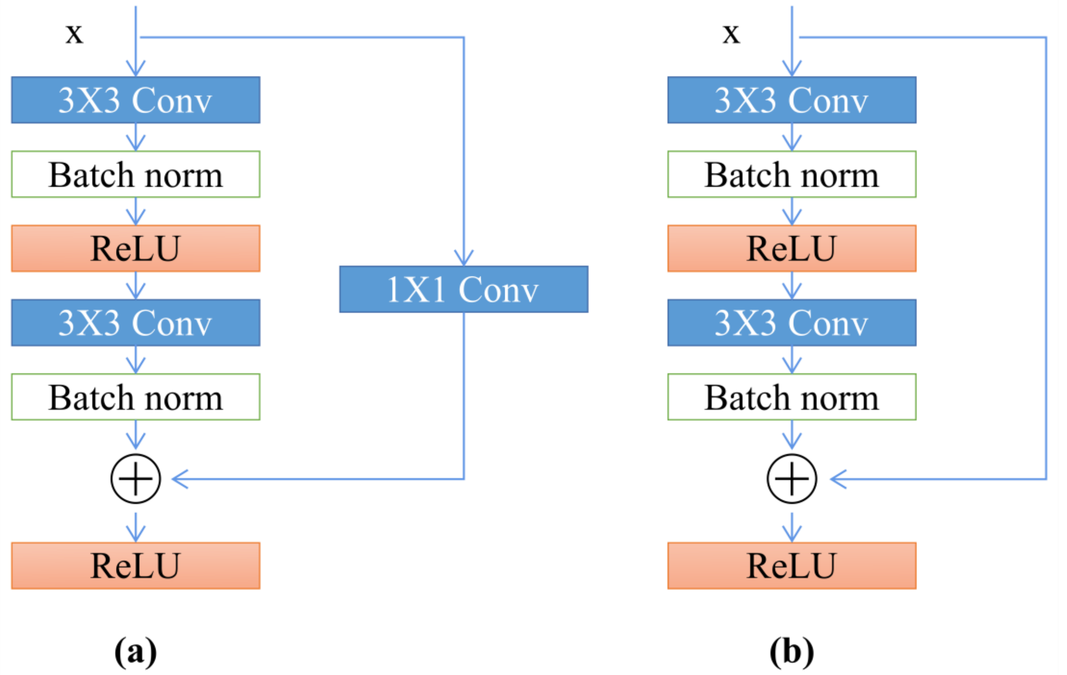

Figure S3. Schematic diagram of (a) Conv Block and (b) Identity Block.
